# Anticancer activity of extracts derived from the mature roots of *Scutellaria baicalensis *on human malignant brain tumor cells

**DOI:** 10.1186/1472-6882-6-27

**Published:** 2006-08-16

**Authors:** Adrienne C Scheck, Krya Perry, Nicole C Hank, W Dennis Clark

**Affiliations:** 1 Neuro-Oncology Research, Barrow Neurological Institute^® ^of St. Joseph's Hospital and Medical Center, Phoenix, AZ 85013, USA; 2 Neurosurgery Research, Barrow Neurological Institute^® ^of St. Joseph's Hospital and Medical Center, Phoenix, AZ 85013, USA; 3 School of Life Sciences, Arizona State University, Tempe, AZ 85287-4501, USA

## Abstract

**Background:**

Flavonoid-rich extracts from the mature roots of *Scutellaria baicalensis *have been shown to exhibit antiproliferative effects on various cancer cell lines. We assessed the ability of an ethanolic extract of *S. baicalensis *root to inhibit the proliferation of malignant glioma cells.

**Methods:**

Cell lines derived from primary and recurrent brain tumors from the same patient and cells selected for resistance to the chemotherapeutic agent 1,3-bis(2-chloroethyl)-1-nitrosourea (BCNU) were used to identify antiproliferative effects of this extract when used alone and in conjunction with BCNU.

**Results and discussion:**

Results indicated that *Scutellaria baicalensis *not only inhibits cellular growth in recurrent and drug resistant brain tumor cell lines, but also demonstrates an increased inhibitory effect when used in conjunction with BCNU.

**Conclusion:**

The results of this study support the efficacy of *S. baicalensis *as an anticancer agent for glioblastomas multiforme and a potential adjuvant treatment to current chemotherapeutic agents used in the treatment of both primary and recurrent GBMs. Further studies of the effects of individual flavonoids alone and in combination with each other and with currently used therapies are needed.

## Background

Malignant gliomas are one of the more lethal forms of cancer. An estimated 18,000 new cases of brain and central nervous system tumors are diagnosed each year and approximately 13,000 people die of their disease in the United States alone [1]. Those diagnosed with the most malignant form of astrocytoma (glioblastoma multiforme, GBM) have a dismal prognosis. The median survival rate of one year has remained essentially unchanged for a number of years despite aggressive treatment regimens that include surgery, radiation and chemotherapy. Complete surgical removal of the tumor is typically not achieved due to the infiltrative nature of these tumors and while radiation and chemotherapy kill the majority of the remaining tumor cells, the rapid recurrence of these tumors suggest the presence within the primary tumor of a subpopulation of cells intrinsically resistant to therapy and capable of survival and growth within the tumor bed following therapy [2,3]. When these tumors recur, they are typically refractory to additional courses of the same therapies. Improvement in the survival and quality of life of glioma patients requires the design of new therapies or therapeutic combinations that are effective and preferably have fewer side effects than those presently available.

One promising new source of therapeutic agents has been discovered in plant secondary metabolites, irregularly occurring compounds that characterize certain plants or plant groups [4]. Recent interest in these secondary metabolites has been focused upon their medicinal properties [5]. For example, flavonoids are a large group of aromatic plant secondary metabolites that are produced in the plant for the purpose of protection from photosynthetic stress, reactive oxygen species (ROS), wounds and herbivores. Studies of flavonoids have produced the most compelling data for the antitumor activities of plant secondary metabolites in various types of cancers [6], and several flavonoids have been shown to inhibit cancer development while exhibiting antioxidant activities in various animal models [7-11]. Furthermore, some studies suggest that the most promising use of these compounds may be as an adjuvant to currently used therapies [12,13].

Numerous cancer research studies have been conducted using traditional medicinal plants in an effort to discover new therapeutic agents that lack the toxic side effects associated with current chemotherapeutic agents. One of the more versatile plants used as a source of flavonoids is the root of the traditional Chinese medicinal herb Baikal skullcap (*Scutellaria baicalensis*), a member of the mint family [14]. Traditionally, the dried roots of *S. baicalensis *were extracted and used in a Chinese herbal medicine "Huang Qin" to treat a variety of ailments [15], and *Scutellaria baicalensis *has remained an important herb in both Chinese and Japanese traditional prescriptions, such as "Xiao-Chai-Hu-Tang" which is used in the treatment of viral hepatitis and a variety of tumors [16-18]. Various flavonoids isolated from this traditional Chinese medicinal plant were shown to have antiandrogenic and growth inhibitory activity against prostate cancer cells *in vitro *and *in vivo *[19-26]. In addition, extracts and isolated flavonoids from this herb have been shown to relieve oxidative stress and immune dysfunction associated with the onset and progress of cancer [8]. Studies have also demonstrated that flavonoids from *S. baicalensis *have the ability to arrest the cell cycle of tumor cell lines that are resistant to multiple chemotherapeutic drugs [27] and act as inhibitors of key steps necessary for the progression of tumor angiogenesis [28].

More recently, *Scutellaria baicalensis *was used as a component of PCSPES, an herbal mixture that showed efficacy in laboratory trials for prostate cancer, small-cell lung cancer and acute myeloid leukemia [29-34]. Despite these promising results in human trials, this herbal mixture was removed from the market due to concerns about contamination [35]. Subsequent work has shown that flavonoids from *S. baicalensis *were likely to have been at least one of the active ingredients in this herbal mixture [31], and *S. baicalensis *extract and its constituents have been shown to cause reduced expression of the androgen receptor and androgen regulated genes in prostate carcinoma [20]. Recent studies have also shown that the flavonoid-rich extract from the roots of *S. baicalensis *exhibit antiproliferative effects upon prostate, squamous, colon, breast, lung, and liver carcinomas, as well as various leukemia cell lines [16,21,36,37]; however, there have been no studies conducted in brain tumors despite suggestions that components of this extract can have effects on microglia in the brain [38]. We, therefore, tested an extract from the root of *S. baicalensis *to determine if it had antiproliferative effects on cells from human malignant brain tumors alone or in combination with 1,3-bis(2-chloroethyl)-1-nitrosourea (BCNU, carmustine), an alkylating agent commonly used in the treatment of human brain tumors.

## Methods

### Plant material extraction

Secondary metabolites, including flavonoids and other phenolic compounds, were extracted from ground mature roots of *S. baicalensis *(common name Huang Qin) using 95% ethanol. Commercially available roots from a California importer of Chinese herbal medicines (Win Hop Fung, Los Angeles) were obtained and ground into a fine powder using a Scientific Apparatus™ soil mill. The root powder was stirred for 24 hours in a 95% ethanol solution in order to extract the secondary metabolites and flavonoids. The crude extract was filtered through a PTFE membrane filter using a Swinney (Millipore Corp., Bedford, MA) filtering device. The extract was dried and quantified for the total amount of crude extract. A stock solution was prepared at 25 mg of solid per ml in absolute ethanol and was further diluted with sterile water immediately before treatment of the cells to achieve a concentration of 10 mg/ml in a 40% (v/v) ethanol solution.

### Cell culture

Cell lines grown from primary and recurrent GBMs from three patients (Table 1) were cultured using previously established protocols [39]. Primary tumors are designated with a 2-letter code (ME, DI) or the last 2 digits of the year followed by a 2-letter code (00WA). The recurrent tumors from the same patient receive the same code with the addition of an "R" (ME/MER, DI/DIR). Following surgery for their primary tumor, both patients from whom we received recurrent tumor samples received 1,3-bis(2-chloroethyl)-1-nitrosourea (BCNU, carmustine) and radiation therapy. Despite therapy, both tumors recurred and the patients underwent additional surgery. Tumor cell line 00WA (Table 1) was selected for use in this study as a low-passage cell line derived from a primary tumor and serial passaged 5 times. Normal glial cells (HJ) grown from a sample obtained from a crainiotomy done for treatment of trauma were also cultured following previously established protocols [39]. All cell lines were cultured in Waymouth's MAB 87/3 (Mediatech, Herndon, Virginia) containing 20% Fetal Bovine Serum (FBS; Intergen Co., Purchase, NY) and incubated at 37°C in a humidified atmosphere of 5% CO_2_. To select for the subpopulation of cells resistant to 10 μg/ml BCNU, cells were treated with gradually increasing concentrations of drug as described [40,41]. Cells were washed with MAB without serum; they were then mock-treated using MAB alone or treated with increasing concentrations of BCNU in MAB (2.5, 5.0, 7.5, 10 μg/ml) for 1 hour at 37°C with 5% CO_2_. Cells were subsequently washed and fed with MAB containing 20% FBS. The cells were treated for three consecutive days then allowed to grow. Treatment was repeated several times until the cells in culture were resistant to 10 μg/ml of BCNU (ME Drug/MER Drug and DI Drug/DIR Drug) as evidenced by the absence of cell death compared to that observed in the mock-treated cultures. Cells were re-treated with 10 μg/ml BCNU every 8–10 passages to maintain the resistant phenotype.

**Table 1:** Patient information

**Tumor code^a^**	**Age^b^**	**Sex**	**Diagnosis^c^**	**Treatment^d^**	**Days between primary and secondary surgery**	**Survival (days)**
DI/DIR	38	F	GBM	Irradiation and BCNU	111	426
ME/MER	49	F	GBM	Irradiation and BCNU	138	285
00WA	39	M	GBM	NA^e^	NA	NAv^f^

^a ^The tumor code is a random 2 letter code. Recurrent tumor from the same patient receives the same code with the addition of R. For each patient the primary and recurrent tumors were diagnosed by the same neuropathologist.

^b ^Age in years at diagnosis of primary tumor.

^c ^GBM = glioblastoma multiforme.

^d ^BCNU = 1,3-bis(2-chloroethyl)-1-nitrosourea.

^e ^NA = not applicable since this was a primary tumor.

^f ^NAv = not available

### AlamarBlue™ metabolic assay

Cells were seeded at a density of approximately 1 – 4 × 10^3 ^cells in 200 μl per well. Following a 24-hour incubation period, cells were treated with 100 μg/ml of *S. baicalensis *extract containing 0.4% ethanol (v/v) in 200 μl of medium. Mock treatment consisted of a 0.4% (v/v) solution of ethanol in 200 μl of medium. Cellular metabolic activity was assayed using AlamarBlue™ (Serotec, Raleigh, NC) and the manufacturer's protocol at 0, 1, 3, 7, 10, and 15 days following treatment. The fluorescent reading was performed using a microtiter plate reader (485 nm λ excitation, 595 λ nm emission).

### Colony forming assay

A Colony Forming Assay (CFA) was used to verify that the cells remaining metabolically active following treatment with *S. baicalensis *extract were also capable of proliferation. Cells were diluted and divided into aliquots containing approximately 1.5 × 10^3 ^cells. Aliquots were treated with a single dose of 0 (untreated), 10, 25, 50, 75, 100, 150 and 200 μg/ml of the extract in MAB 87/3 medium supplemented with 20% FBS for 1 hour. Following incubation, cells were washed three times with fresh medium. Cells were resuspended in the medium and aliquots of 1.5 ml containing approximately 2 × 10^3 ^cells were dispensed into three 60 mm culture dishes. Cells were cultured for at least 6 divisions, approximately 14 days, before they were fixed with methanol and stained with 4% Giemsa stain. Colonies, defined as 50 or more cells, were counted and plotted as a percentage of the control (untreated) colonies.

### Trypan blue exclusion cell viability assay

The cells were plated at approximately 1 × 10^5 ^cells per well in 6-well tissue culture plates in 2 ml of medium and incubated at 37°C at 5% CO_2_. After 24 hours, the medium was removed and replaced with fresh medium plus 20% FBS and supplemented with *S. baicalensis *extract (0, 25, 50, 100, 150, and 200 μg/ml) or a combination of *S. baicalensis *extract and BCNU (2.5 or 5 μg/ml). Cells were harvested 48 hours after treatment by digestion with 0.25% trypsin-EDTA solution at 37°C for 2–3 minutes. The cells were stained with trypan blue and live cells were enumerated. Cell counts were expressed as mean ± standard deviation (SD).

### Statistical analysis

All results are expressed as mean ± standard deviation (SD). Statistical differences between correlated samples were evaluated using Student's *t*-test and noted to be significantly different where p < 0.05. Student *t*-test calculations were assessed on the VassarStats Statistical Computation website [42]. Composite treatments were compared using one-way analysis of variances (ANOVA) and considered significantly different where probability values were found to be equal to or less than 0.05. All ANOVA tests, as well as mean and SD calculations, were performed using GraphPad Prism (GraphPad Software, Inc., San Diego, USA).

## Results

### Effects of *S. baicalensis *on glioma cell metabolic activity

**Figure 1:**
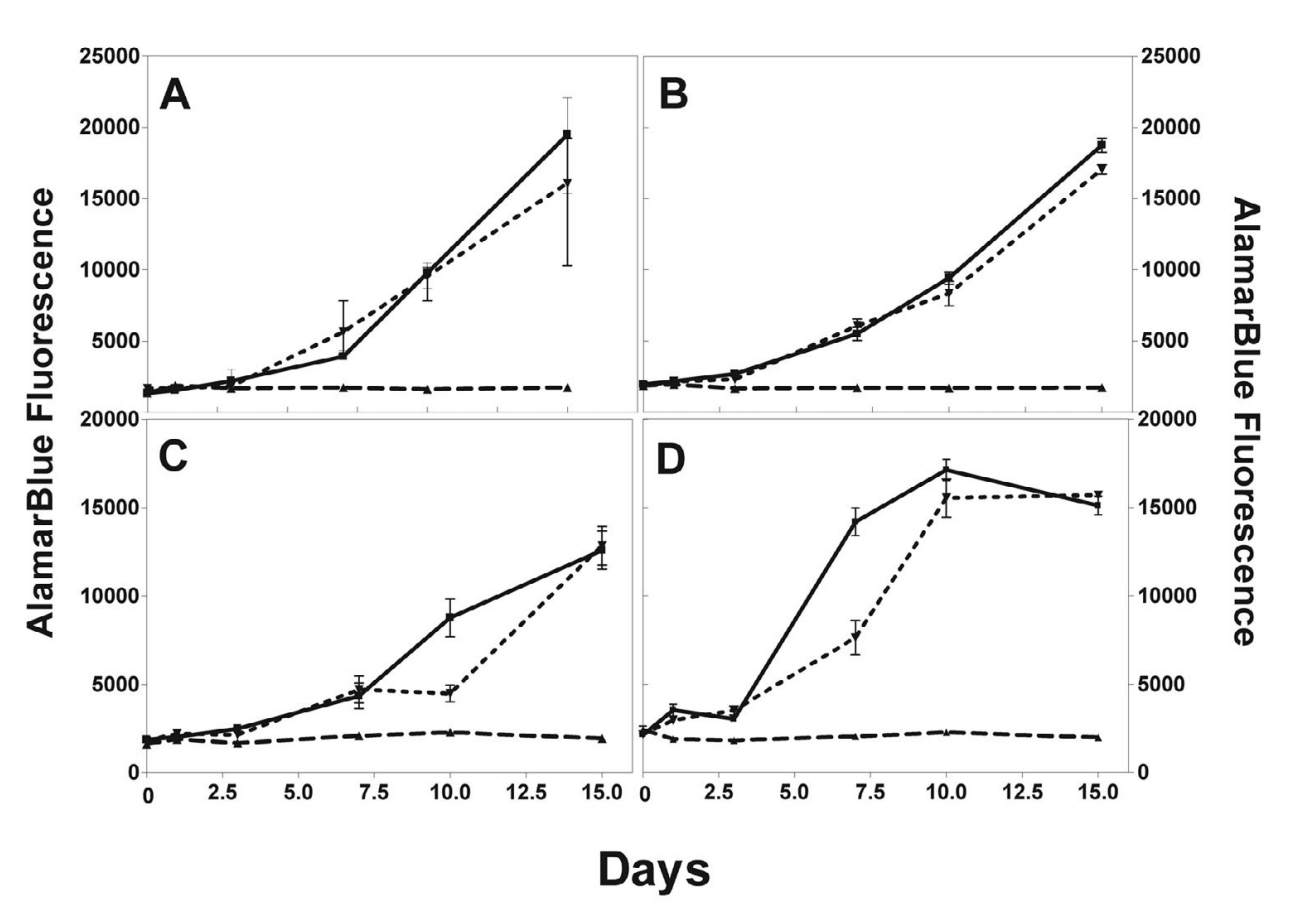
Time course treatment of ME series cells with *S. baicalensis *extract. Metabolic activity was measured using AlamarBlue™. Wells were subconfluent on Day 0. Cells were left untreated (■-■), mock treated with 0.4% ethanol (∇-∇) or treated with a 0.4% ethanol solution containing 100 μg/ml *S. baicalensis *extract (△-△). Data is mean ± SD using 5 replicates. (A) cells from primary tumor (ME), (B) ME cells selected for resistance to 10 μg/ml BCNU (ME drug), (C) cells from recurrent tumor (MER), (D) cells from recurrent tumor selected for resistance to 10 μg/ml BCNU (MER drug).

The metabolic inhibitory effects of an extract from *S. baicalensis *were studied in cell lines cultured from paired primary (ME) and recurrent (MER) glioblastoma multiforme (GBM) tumors obtained from a single patient, as well as primary and recurrent cells that have been selected *in vitro *for resistance to 10 μg/ml of BCNU (ME Drug/MER Drug). Metabolic activity was assessed at 0, 1, 3, 7, 10, and 15 days following treatment with 100 μg/ml of the extract using an AlamarBlue metabolic assay. All wells were subconfluent when the experiment was begun to allow for changes in cell growth. In this way, we would see a difference regardless of whether the effect of the extract was cytotoxic or cytostatic. There was a significant (p < .05; n = 5) difference in the metabolic activity exhibited by cells treated with *S. baicalensis *extract compared with mock- or ethanol treated cells. There was no increase in metabolic activity exhibited in the ME and MER glioma cell lines, as well as subpopulations of these cell lines that were selected for BCNU resistance (ME Drug and MER Drug) for the 15 day duration of the experiment (Figure 1), while the untreated glioma cells (controls) exhibited exponential increases in metabolic activity. As an additional control, glioma cells were treated with a 0.4% (v/v) ethanol solution (solvent vehicle). Although there was some difference in fluorescence between untreated and ethanol treated MER cells at day 7 (Figure 1D) and 10 (Figure 1C), there was good growth in all four cell lines and the ethanol-treated cells showed no significant decrease in metabolic activity compared to the untreated control cells by the fifteenth day of culture. Microscopic examination showed extensive cytopathic effect (CPE) in cells treated with the *S. baicalensis *extract (data not shown).

### Effects of *S. baicalensis *on glioma cell viability and proliferation

**Figure 2:**
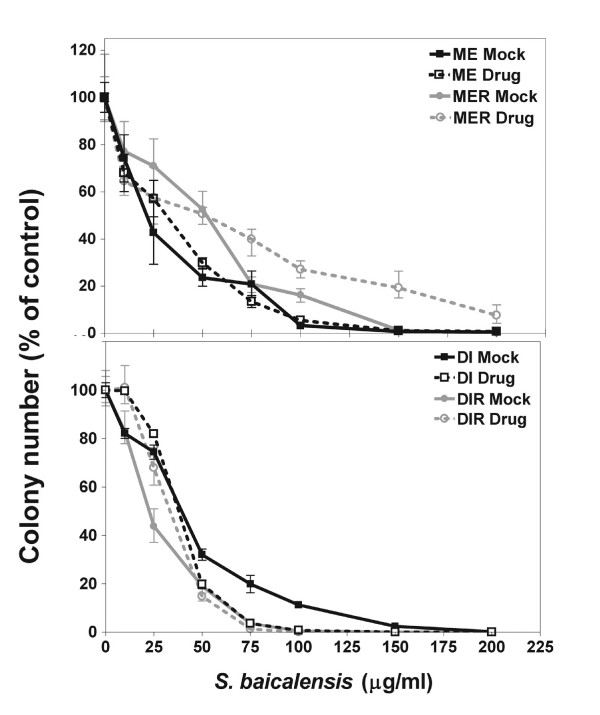
Dose-dependent inhibition of glioma cell proliferation by *Scutellaria baicalensis *extract. Glioma cell lines from primary tumors (ME and DI) and recurrent (MER and DIR) tumors, as well as BCNU resistant (designated "Drug") glioma cell lines were cultured with various concentrations of *S. baicalensis *extract (0–200 μg/ml). After treatment, cells were incubated for 14 days to allow for colony growth. Colonies of at least 50 cells were counted using a 4% Giemsa stain. Results were normalized to the control (untreated cells). Data show means ± SD of 3 replicates.

Colony forming assays were done to demonstrate a dose response to the *S. baicalensis *extract and to show that the effect is cytopathic and not cytostatic. Cells from the paired primary and recurrent tumors ME/MER and DI/DIR prior to and following selection for resistance to 10 μg/ml BCNU were treated with 0 – 200 μg/ml *S. baicalensis *extract as described in the methods section. Cells were allowed to grow to form colonies of at least 50 cells. As seen in Figure 2, cells from all 8 cell lines showed a dose-dependent response to the *S. baicalensis *extract.

**Figure 3:**
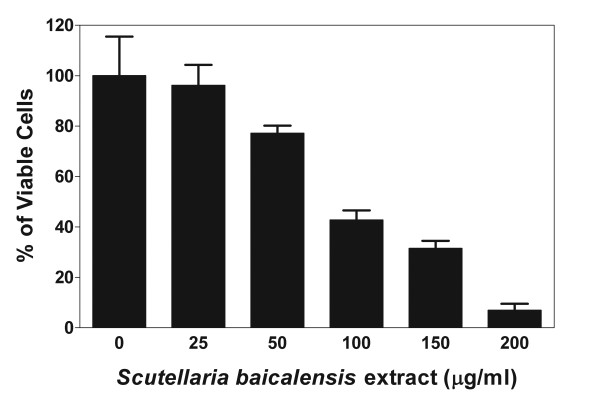
Dose-dependent growth inhibition of a low-passage glioma cell line by an extract from *S. baicalensis*. The viability of cell line 00WA following treatment with 0–200 μg/ml of *S. baicalensis *extract was tested by trypan blue exclusion at serial passage 5. Results are the mean ± SD of 2 replicates and data were normalized to the control.

To demonstrate that the effect of the *S. baicalensis *extract on glioma cells was not a result of long-term *in vitro *cultivation of the cells, we tested the extract on cell line 00WA after 5 serial passages *in vitro*. This was the minimum number of passages required to obtain enough cells to perform the assay. Cells were treated with varying concentrations of the *S. baicalensis *extract as described in the Methods section, and the percentage of viable cells was determined by trypan blue exclusion 48 hours later. Figure 3 shows that the extract caused a dose-dependent reduction in viability similar to that observed in the ME/MER and DI/DIR cell line series.

### Effects of *S. baicalensis *on normal glial cells

**Figure 4:**
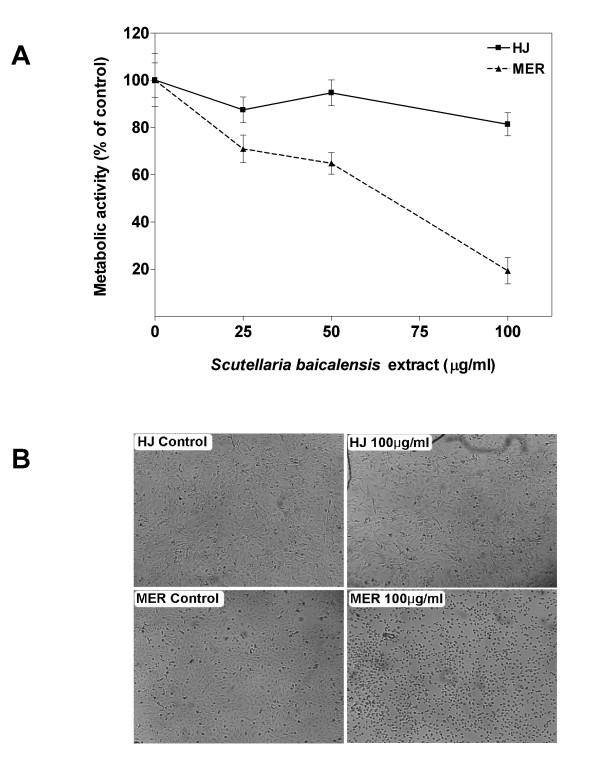
The cytotoxic effects of *Scutellaria baicalensis *extract on normal glial cells and glioma cells. Wells were essentially confluent at the beginning of the experiment. (A) Metabolic activity of normal glial cells (HJ) and cells from a recurrent tumor (MER) were measured using the AlamarBlue™ metabolic assay. Glioma cells were cultured with 100 μg/ml of the *S. baicalensis *extract for approximately 48 hours. Results were presented as a percentage of the control (untreated). Data show means ± SD of 3 replicates. (B) Photomicrographs of cells 48 hours following treatment with 100 μg/ml of *S. baicalensis *extract.

To assess whether the extract demonstrates toxicity in normal cells, normal human glial cells (HJ) were plated and allowed to grow until they were close to confluence. These cells were then cultured in the presence and absence of various concentrations of the extract for 48 hours. As shown in Figure 4, normal glial cells treated with 25–100 μg/ml of *S. baicalensis *extract for 48 hours displayed no significant change (p > .05; n = 3) in metabolic activity assayed using AlamarBlue and compared to the control (untreated) cells. For comparison, a recurrent glioma cell line (MER) was also treated with 25-100 μg/ml of the extract for 48 hours. The glioma cells treated with *S. baicalensis *extract at concentrations of 50 and 100 μg/ml displayed a significant reduction (p < .05, n = 3) in metabolic activity when compared to the metabolic activity of the control (untreated) cells.

Microscopic examination of the cells following treatment with 100 μg/ml of the extract showed marked differences in the normal glial cells compared to the tumor cells (Figure 4B). The MER culture demonstrated an increase in the number of detached cells floating in the medium. The floating cells exhibited atypical morphologies such as DNA condensation, a hallmark of cell death (Figure 4B). The normal glial (HJ) cells did not show a substantial increase in floating cells or cells with DNA condensation or other hallmarks of cell death.

### Combination treatment with *S. baicalensis *and BCNU

**Figure 5:**
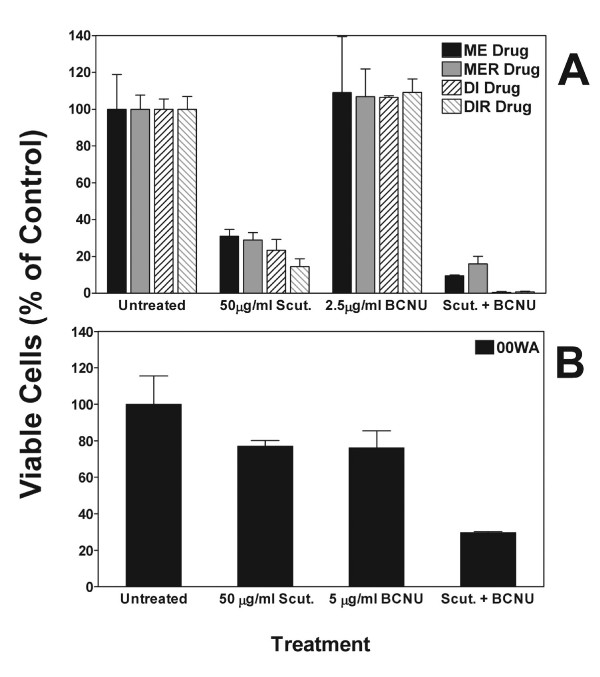
The effects of *Scutellaria baicalensis *extract (50 μg/ml) and BCNU on glioma cell viability in BCNU-resistant and low passage cell lines. (A) Glioma cell lines from primary and recurrent tumors (ME/MER; DI/DIR) selected for resistance to 10 μg/ml BCNU (designated "Drug") were treated with either 50 μg/ml of *S. baicalensis *extract alone (Scut.), 2.5 μg/ml of BCNU alone, or a combination of the *S. baicalensis *extract and BCNU (Scut. + BCNU). (B) Low passage cells from tumor 00WA were treated with either 50 μg/ml of *S. baicalensis *extract alone (Scut.), 5 μg/ml of BCNU alone, or a combination of the *S. baicalensis *extract and BCNU (Scut. + BCNU). The percentage of viable cells following 3 consecutive days of treatment was assessed using a trypan blue exclusion assay. Results are normalized to the control (untreated) cells and data is the mean ± SD for 2 replicates.

**Figure 6:**
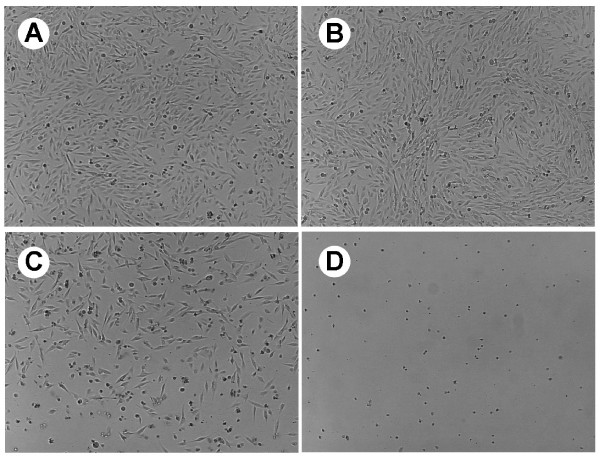
Photomicrographs of ME Drug cells (selected for resistance to 10 μg/ml BCNU) treated with either (B) 5 μg/ml BCNU, (C) 50 μg/ml of *S. baicalensis *extract, (D) or a combination of *S. baicalensis *extract and BCNU. Glioma cells were treated for 3 consecutive days and then compared to the (A) control (untreated) cells.

To determine if the *S. baicalensis *extract could potentiate the effects of conventional chemotherapeutic agents, we tested the effect of combined treatment using 50 μg/ml of *S. baicalensis *extract and 2.5 μg/ml of BCNU on the viability of cells from primary and recurrent cells selected for resistance to 10 μg/ml BCNU (Figure 5A). As expected, 2.5 μg/ml of BCNU alone had no effect on these cells and 50 μg/ml of *S. baicalensis *extract reduced viability to 15–30% of control; however, when used in combination survival was reduced substantially, particularly in the DI and DIR cell lines. The differences between the individual treatments and the combined treatment were found to be significant in all but the ME drug cells by one-way ANOVA (ME Drug: P = 0.0557 F = 8.785; MER Drug: P = 0.0116 F = 27.77; DI Drug: P = 0.0005 F = 253.3 and DIR Drug: P = 0.0010 F = 144.1). Fewer than 5% of any of the cells survived when the BCNU dose was increased to 5 μg/ml, even though this dose alone caused little, if any, CPE (data not shown). Microscopic examination of the combined effects of BCNU and the *S. baicalensis *extract again indicated an increase in the number of detached (floating) cells with atypical morphologies when compared to cells treated with either BCNU or the extract alone (Figure 6).

The effect of combined treatment on low passage, BCNU naive 00WA cells showed a similar significant potentiation of the drug effect, although the overall effect was less than that observed with the ME and DI cell series (Figure 5B). BCNU or *S. baicalensis *extract alone reduced viability to 75%; however, combination treatment resulted in a reduction of viability to 30% of the control, mock-treated cells (p = 0.0141 F = 24.20 by one-way ANOVA).

## Discussion

Treatment of malignant brain tumors typically includes surgery, radiation and chemotherapy; however, this tumor typically recurs following therapy and the recurrent tumor is often refractory to additional therapy. The identification of a novel therapy that is effective against recurrent tumor could substantially impact the morbidity and median survival of patients with this disease. The purpose of this study was to investigate the effects of plant secondary metabolites from *Scutellaria baicalensis *on cells from primary and recurrent gliomas prior to, and following, selection for therapy resistance.

There are many reports of the medicinal utility of the Chinese herb *S. baicalensis *for the treatment of a host of diseases. Although there have been numerous studies on the effects of *S. baicalensis *extracts and isolated flavonoids on cancer cells, few have studied their effects on glioma cells. Further, there are no studies examining the effect of extracts of this herb on cells from tumors that have recurred following standard therapies. We, therefore, tested an ethanol extract from *S. baicalensis *for its ability to inhibit the growth of cells from primary gliomas, cells from recurrent gliomas from the same patient and cells selected for resistance to BCNU therapy *in vitro*. We also tested cells cultured from primary gliomas after only 5 serial passages *in vitro*. Our work demonstrated that the *S. baicalensis *extract caused a dose-dependent inhibition of growth of all of these cell lines, irrespective of whether they were from a primary or recurrent tumor. Reduced metabolic activity due to the *S. baicalensis *extract was demonstrated using AlamarBlue™, and cytotoxicity was demonstrated by colony forming assays as well as by trypan blue exclusion and microscopic examination. A dose of 50 μg/ml of the extract typically reduced the population of glioma cells by at least 50% of the control (untreated) population; however, the same dose of extract had little, if any, effect on HJ cells, the cultured normal glia. In fact, 25 – 100 μg/ml of *S. baicalensis *extract did not induce the same inhibitory effects in HJ cells that were observed in all of the glioma cells tested. The normal glial cells also failed to undergo the typical morphological changes seen following treatment of the glioma cells. This suggests that the *S. baicalensis *extract may not affect normal cells to the extent that it affects tumor cells, thus warranting *in vivo *studies.

The effect of the *S. baicalensis *extract on cells from recurrent tumor is of particular interest. Recurrent tumor generally arises from cells in the primary tumor that survived treatment with radiation and chemotherapy; thus, recurrent tumor is often refractile to further therapies. Our data demonstrates that not only are the cells from recurrent tumor sensitive to the effects of the *S. baicalensis *extract, but cells from primary and recurrent tumor selected for resistance to 10 μg/ml BCNU are sensitive to lower doses when given in combination with *S. baicalensis *extract. This suggests that this extract may have its greatest utility when used in combination with currently available therapies. This work is similar to previous studies showing that *S. baicalensis *extracts may reduce tumor growth and proliferation when applied to chemotherapy and radiation resistant tumors in various forms of cancer [18,43-45]. One postulated mechanism for this is the inhibition of extracellular signal-regulated kinase (ERK). Pathways involving ERK are activated in most GBMs [46], and inhibition of ERK has been shown to inhibit growth of GBMs and medulloblastomas alone and in combination with temozolomide [47,48].

The effects of extracts and isolated flavonoids are not all antiproliferative. Choi et al [49] found that an aqueous extract from *S. baicalensis *reduced the apoptotic death induced in neuronal HT-22 cells exposed to H_2_O_2 _by increased Bcl-2 and reducing Bax levels. In addition, baicalein, a major component of the *S. baicalensis *extract, can exert either pro- or anti-apoptotic activity depending on the cell type. For example, in addition to its antiproliferative effects, baicalein has been shown to prevent the loss of viability and apoptosis induced in the human glioma cell line A172 by cisplatin [50]. Regardless of this, our data has demonstrated a potential role for the use of *S. baicalensis *as an adjuvant therapy in the treatment of human malignant brain tumors, particularly recurrent tumors.

## Conclusion

In summary, the results of this study support the efficacy of *S. baicalensis *as an anticancer agent for glioblastomas multiforme and a potential adjuvant treatment to current chemotherapeutic agents used in the treatment of both primary and recurrent GBMs. Further studies of the effects of individual flavonoids alone and in combination with each other and with currently used therapies are in progress.

## Abbreviations

1,3-bis(2-chloroethyl)-1-nitrosourea (BCNU, carmustine)

glioblastoma multiforme (GBM)

*Scutellaria baicalensis *(*S. baicalensis*)

colony forming assay (CFA)

cytopathic effect (CPE)

## Competing interests

The author(s) declare that they have no competing interests.

## Authors' contributions

This work was done in partial fulfillment of the requirements for a Master's Degree by KP. ACS and WDC conceived the study and directed its design and coordination. WDC provided the materials for the *S. baicalensis *extract and directed its preparation. ACS and NCH provided the glioma cells and directed their use and chemotherapy resistance testing. KP did the cell culture and all resistance assays. All authors read the manuscript and agreed to its contents.

## Pre-publication history

The pre-publication history for this paper can be accessed here:


